# Levels, severity, and determinants of stunting in children 0–59 months in Afghanistan: Secondary analysis of Multiple Indicator Cluster Survey, 2022-23

**DOI:** 10.1371/journal.pgph.0004423

**Published:** 2025-04-08

**Authors:** William Joe, Atma Prakash, Said M. Yaqoob Azimi, Melanie Galvin, Zivai Murira, Gustavo Nicolas Paez Salamanca, Vani Sethi

**Affiliations:** 1 Institute of Economic Growth, Delhi, India; 2 UNICEF Afghanistan, Kabul, Afghanistan; 3 UNICEF Regional Office for South Asia, Kathmandu, Nepal; PLOS: Public Library of Science, UNITED STATES OF AMERICA

## Abstract

Childhood stunting is a critical nutritional concern for Afghanistan. Prioritizing development assistance toward child nutrition requires recent estimates on child stunting and timely insights on determinants at national and sub-national levels. This study addresses this gap by estimating the prevalence and determinants of stunting and severe stunting in children under-five using the latest publically available data. The recent wave of Afghanistan Multiple Indicator Cluster Survey (MICS 2022-23) was analyzed to estimate the prevalence of stunting (height-for-age Z-score <-2SD) and severe stunting (<-3SD) by demographic and socioeconomic characteristics. The predictors of stunting and severe stunting outcomes were examined using multivariate logistic regression analyses with four domains of independent variables - child, maternal, and household characteristics and complementary feeding practices. In Afghanistan, 44·5% of children were stunted and 21.6% were severely stunted. The southern region has the highest burden of stunting (55%). Under-five females were less likely to be stunted than males [OR 0·89, 95% CI (0·84, 0·95)]. The likelihood of stunting increased with age of the child. Lack of maternal education, lower wealth quintiles, no exposure of the mother to mass media, and poor dietary diversity were the key predictors of stunting. Determinants of severe stunting mirrored those of stunting, with the additional risk for 24-59 months age group and higher birth order. Socioeconomic status, maternal education, child age, birth order, dietary practices, and geographical location were key determinants of stunting. Targeted interventions addressing poverty, education for women, family planning, and improved nutrition are crucial to reducing childhood stunting in Afghanistan.

## Introduction

Afghanistan’s geopolitical environment, macroeconomic instabilities, and frequent natural calamities adversely affect the already fragile food, livelihood, and nutrition security of the populace [1]. In 2022, 10 percent of children (0-59 months) suffered from acute malnutrition (wasting, low mid-upper arm circumference or oedema) [2]. In 2023, several reports highlighted depleted household food stocks, particularly in the northern areas and highlands and increased unemployment [3,4]. While development assistance continued in the form of humanitarian food and cash transfers to enable households to meet their survival needs, enhancing life-saving nutrition service delivery points (mobile and fixed health facilities) provide both prevention and screening and lifesaving treatment services for children suffering from severe wasting [5,6].

A critical constraint to tailoring food and nutrition interventions in Afghanistan was the limited availability of recent, disaggregated data on child nutritional status. The Afghanistan Multiple Indicator Cluster Survey (MICS) 2022-2023 partially addressed this data gap [7]. However, a more granular analysis was necessary to inform program development. This study leverages a secondary analysis of the MICS data to estimate the prevalence and examine the determinants of stunting and severe stunting among children under-five (categorized by age groups: 0-23 months, 24-59 months, and 0-59 months) at the national, regional, and provincial levels. Stunting reflects the cumulative impact of poverty and deprivation on a child’s growth, a persistent challenge for many Afghan children. The findings aim to inform the development of targeted food and nutrition assistance programs to prevent childhood stunting and intervention for affected households.

### Data and methods

We used publicly available and anonymized data from the Afghanistan Multiple Indicator Cluster Survey-6 (MICS-6) [7]. Funded by UNICEF and the World Bank, MICS-6 data collection (September 2022-February 2023) captured the post-pandemic, drought, earthquake, and economic shock context in Afghanistan. The representative sample yielded provincial estimates for all 34 provinces. Four standardized English CAPI questionnaires, customized and translated into Dari and Pashto, were used: a) Household questionnaire gathered demographic information on household members, the dwelling, and the household itself; b) Women’s questionnaire (ages 15-49) was administered to all women in each household; c) Under-five questionnaire was administered to mothers/caretakers of all children under-five in the household; d) Children’s questionnaire (ages 5-17) was administered to the mother/caretaker of one randomly selected child aged 5-17 in the household.

The National Statistics and Information Authority and UNICEF Afghanistan led the survey, adhering to the global MICS program’s standard technical framework. Detailed methodology and datasets are available in the UNICEF Afghanistan MICS report [7]. Questionnaires 1-3 (households, women aged 15-49, and under-five children) from the Afghanistan MICS 2022-2023 were used for the analysis. While the dataset included multiple records per child under five (n=33,398), focusing on stunting (height-for-age), only those with complete age and height data were initially considered. We further excluded records lacking data on maternal characteristics (age, fertility, education) or household characteristics (assets, water/sanitation, household size). The final sample comprised 31,189 children under five, disaggregated into subsamples by age: 12,055 children aged 0-23 months and 19,134 children aged 24-59 months (S1 Fig).

This study employed two binary dependent variables: stunting and severe stunting. According to the WHO Multicentre Growth Reference Study Group [8], stunting was indicated by a height-for-age Z-score (HAZ) below two standard deviations of the reference median (<-2SD), while severe stunting was indicated by HAZ <-3SD. Stunting variable thus includes both moderate as well as severe stunting. Independent variables, selected based on existing literature [9,10], were categorized into four domains: child characteristics, maternal characteristics, household characteristics, and infant and young child feeding (IYCF) practices. The specific variables are as follows:

- Child characteristics: Age in months (and squared term), sex, and birth order.
- Maternal characteristics: Current age (and squared term), formal education level (no education, primary, secondary, higher,), exposure to at least one media source (newspaper, television, mobile, internet), and Preceding birth interval (first child, less than 2, and two or more).
- Household characteristics: Location (urban/rural, province, region), number of family members, access to improved water and sanitation, and wealth index as provided in the MICS data set (poorest, poor, middle, rich, and richest) [7].
- IYCF practices:
  - For children under six months: Early initiation of breastfeeding (within 1 hour), The exclusive breastfeeding indicator is specified for the first three days after birth as opposed to the conventional two days in the MICS 2022-23,(11) and exclusive breastfeeding in the last 24 hours.
  - For children 6-23 months: Early initiation of breastfeeding, exclusive breastfeeding for 3 days and for first 6 months, minimum meal frequency, minimum acceptable diet and minimum dietary diversity (consuming foods from at least 5 of 8 WHO/UNICEF food groups) [11].

To assess the bivariate association of the four domains of independent variables with the two outcome variables (any stunting and severe stunting), we ran domain-wise cross-tabulations for independent variables with each outcome variable and used Pearson’s chi-squared test for significance based on normality assumptions. We also conducted the non-parametric Wilcoxon rank-sum test and the Tukey honest significant difference (HSD) test to measure the outcome difference based on the cases of two categories and multiple categories of correlates, respectively. A logistic regression model was chosen based on the lowest Bayesian Information Criteria (BIC) score compared to linear probability and probit regression models. Separate models were estimated for the combined 0-59 months as well as with age-based stratifications (0-5 months, 6-11 months, 12-23 months, and 24-59 months). Stratified analysis was also conducted based on educational status of the mother (educated vs uneducated with no formal schooling) as well as household wealth quintile (top 40% vs bottom 60%). Regression coefficients were presented as Odds Ratios (OR) with 95% Confidence Intervals (CI) due to the dichotomous nature of the dependent variables (stunting and severe stunting). Additionally, sensitivity analysis was conducted by including the age of the child and mother (and their squared terms) as continuous variables and by employing stepwise regression to assess variable selection consistency. All analyses were performed using Stata 18·0 software.

### Ethics committee

Given the utilization of a secondary dataset, the Ethics Committee of the Institute of Economic Growth deemed the study exempt from requiring Ethical Approval.

## Results

Of the 31,189 children aged 0-59 months, there was a near-equal distribution by sex (51% boys, 49% girls). The majority of these children (77%) resided in rural areas. Notably, more than 60 percent of the children had a preceding birth interval of two or higher (Table 1). Three percent of the mothers were below 18 years of age. Furthermore, a significant disparity in maternal education and media exposure was evident. Seventy-nine percent of mothers lacked formal schooling, and 70% had no access to newspapers, radio, television, or the Internet. Household characteristics indicated that improved drinking water facilities were present in 73% of households, while improved sanitation facilities were found in 47% of households.

**Table 1:** Sample distribution of children by age group categories and domain (child, maternal, household, and IYCF) characteristics, Afghanistan (MICS 2022-23).

Background characteristics	Age (0 to 6 months)		Age (6 to 11 months)		Age (12 to 23 months)		Age (24 to 59 months)		Age (0 to 59 months)	
	N	(%)	N	(%)	N	(%)	N	(%)	N	(%)
**Gender**										
Female	1547	47.7	1470	48.5	2909	49.5	9355	48.6	15281	48·7
Male	1627	52.3	1538	51.5	2964	50.5	9779	51.4	15908	51·3
**Age in months**										
0 to 5									3174	10·2
6 to 11									3008	9·8
12 to 17					3157	52.2			3157	10·2
18 to 23					2716	47.8			2716	9·3
24 to 36							6639	34.8	6639	21·1
37 to 59							12495	65.2	12495	39·4
**Birth order**										
First	560	17.8	464	16.4	966	15.7	3015	16.2	5005	16·3
2 to 3	971	30.9	879	29.5	1776	30.7	5795	30.3	9421	30·3
4 or more	1643	51.3	1665	54.2	3131	53.6	10324	53.6	16763	53·4
**Preceding birth interval**										
First child	567	18.2	472	16.6	980	15.9	3040	16.3	5059	16·5
<2	675	19.6	684	22.1	1547	24.3	6077	30.6	8983	27·4
>=2	1932	62.2	1852	61.2	3346	59.8	10017	53.1	17147	56·1
**Age of mother (In Years)**										
15 to 19	241	8.8	157	6.2	249	4.4	234	1.4	881	3·2
20 to 29	1791	55.3	1603	53.4	3197	54.7	9041	48.6	15632	50·9
30 to 39	949	30.5	1020	33.7	1947	33.1	7426	38.4	11342	36·1
40 to 49	193	5.4	228	6.7	480	7.8	2433	11.6	3334	9·7
**Mother educational attainment**										
Uneducated	2541	76.8	2373	75.9	4715	75.8	16085	80.1	25714	78·5
Educated	633	23.2	635	24	1158	24.2	3049	19.9	5475	21.5
**Marital status**										
Currently Married	3163	99.7	3001	99.8	5832	99.1	18867	98.5	30863	98·9
Widowed/Divorced/Separated	11	0.3	7	0.2	41	0.9	267	1.5	326	1.1
**Exposure to mass media**										
Yes	811	29.3	827	29.6	1560	31.6	4718	29.2	7916	29.7
No	2363	70.7	2181	70.4	4313	68.4	14416	70.8	23273	70.3
**Place of Residence**										
Urban	480	22.1	476	23.1	930	23.9	2829	22.2	4715	22·6
Rural	2694	77.9	2532	76.9	4943	76.1	16305	77.8	26474	77·4
**Household wealth index quintile**										
Poorest	658	20	622	20.1	1345	20.8	4634	22.2	7259	21·5
Poor	740	21.5	715	20.7	1294	19.9	4651	21.8	7400	21·3
Middle	737	20.4	676	21.6	1342	20.6	4174	20.1	6929	20·4
Rich	622	21.4	593	20.2	1096	19.4	3380	18.9	5691	19·4
Richest	417	16.8	402	17.5	796	19.3	2295	17	3910	17·4
**Improved drinking water facility**										
Yes	2190	73.9	2124	76.4	3975	73.6	12745	72.8	21034	73·4
No	984	26.1	884	23.6	1898	26.4	6389	27.2	10155	26·6
**Improve toilet facility**										
Yes	1555	46	1484	47.4	2860	47.5	9178	46.4	15077	46·7
No	1619	54	1524	52.6	3013	52.5	9956	53.6	16112	53·3
**Open defecation**										
Yes	706	21.4	655	20.1	1271	20.2	4366	21.3	6998	21
No	2468	78.6	2353	79.9	4602	79.8	14768	78.7	24191	79
**Region**										
Central	1100	28.7	1107	29.3	2102	30.6	6671	29.9	10980	29·8
East	447	12.2	403	11.1	732	9.7	2675	10.8	4257	10·8
North	768	28.9	713	28.7	1469	29.1	4595	28.5	7545	28·7
South	553	17.8	511	17	948	16	3113	16.3	5125	16·5
West	306	12.4	274	14	622	14.6	2080	14.5	3282	14·2
N	3174	100	3008	100	5873	100	19134	100	31189	100

Breastfeeding practices and dietary diversity in children aged 0-23 months revealed high ever-breastfeeding rates (96·6%) but suboptimal initiation practices (47·9% initiated within one hour). 61·7% of the children were exclusively breastfed for the first three days after birth, whereas 62·1% of the infants (0-5 months) were fed exclusively with breast milk during the previous day. However, continuation rates declined in children aged 12-23 months. Only 14·8% of children (6-23 months) fulfill minimum dietary diversity norms of consuming food and beverages from at least five of eight defined food groups during the previous day. Furthermore, there was a low consumption of fruits and vegetables (60·7% with no intake) and animal-source protein (24·4% consuming eggs or meat), whereas only 7·3% of children achieved a minimum acceptable diet (Table 2).

**Table 2:** Infant and Young Child Feeding Practices, 0-23 months, Afghanistan (MICS 2022-23).

Indicators		Number (n)	Percentage (%)	Sample size (N)
Ever breastfed	Children 0-23 months who were ever breastfed	11605	96·6	12055
Early initiation of breastfeeding	Children 0-23 months who were put to the breast within one hour of birth	5423	47·9	12055
Exclusively breastfed for the first two days after birth	Children 0-23 months who were fed exclusively with breast milk for the first three days after birth*	7665	61·7	12055
Exclusive breastfeeding under six months	Infants 0–5 months of age who were fed exclusively with breast milk during the previous day	1983	62·1	3174
Mixed milk feeding under six months	Infants 0–5 months of age who were fed formula and/or animal milk in addition to breast milk during the previous day	454	15	3174
Continued breastfeeding 12–23 months	Children 12–23 months of age who were fed breast milk during the previous day	4033	68	5873
Introduction of solid, semisolid or soft foods 6–8 months	Infants 6–8 months of age who consumed solid, semi-solid or soft foods during the previous day	879	58	1599
Minimum dietary diversity 6–23 months	Children 6–23 months of age who consumed foods and beverages from at least five out of eight defined food groups during the previous day	1299	14·8	8881
Minimum meal frequency 6–23 months	Children 6–23 months of age who consumed solid, semi-solid or soft foods (but also including milk feeds for non-breastfed children) the minimum number of times or more during the previous day	2915	35·4	8881
Minimum milk feeding frequency for non-breastfed children 6–23 months	Non-breastfed children 6–23 months of age who consumed at least two milk feeds during the previous day	878	39·7	2109
Minimum acceptable diet 6–23 months	Children 6–23 months of age who consumed a minimum acceptable diet during the previous day	642	7·3	8881
Egg and/or flesh food consumption 6–23 months	Children 6–23 months of age who consumed egg and/or flesh food during the previous day	2221	24·4	8881
Zero vegetable or fruit consumption 6–23 months	Children 6–23 months of age who did not consume any vegetables or fruits during the previous day	5464	60·7	8881
Bottle feeding 0–23 months	Children 0–23 months of age who were fed from a bottle with a nipple during the previous day	3219	27	12055

*While the WHO (2021) definition is two days, AFGH MICS 2022-23 used three days. Note: Although WHO (2021) also recommends indicators for (i) Sweet beverage consumption 6–23 months Children 6–23 months of age who consumed a sweet beverage during the previous day and (ii) Unhealthy food consumption 6–23 months: Children 6–23 months of age who consumed selected sentinel unhealthy foods during the previous day. The AFGH MICS 202-23 did not collect this information, hence this information is missing.

Analysis of under-five children (0-59 months) revealed a prevalence of stunting at 44·5% (Table 3). This prevalence differed significantly between males (45·5%) and females (43·5%) (p<0·0001, S1 Table). Notably, the male-female disparity persisted across all age groups (0-5 months, 6-23 months, and 24-59 months). Stunting was present in 26·4% of children aged 0-5 months, doubling after two years of age. Children with short inter-pregnancy intervals (<2 years) exhibited significantly higher stunting rates (52·2%) compared to firstborns (43·8%) or children with longer intervals (>2 years) (41·0%) (p<0·0001, both Pearson chi-square and Tukey HSD tests, S2 Table). Stunting was alarmingly high (73·8%) among children aged 24-59 months whose mothers were under 19 (p<0·0001, Pearson chi-square test). Maternal education level also significantly impacted stunting prevalence (p<0·0001, Tukey HSD test). Children of mothers with no formal education had a substantially higher prevalence (47·5%) of stunting compared to those with higher education (28·7%). Finally, exposure to mass media played a role, with lower stunting rates observed in children whose mothers had at least weekly exposure (35·1%) compared to those without exposure (48·5%).

**Table 3:** Prevalence of stunting and severe stunting among children by age group categories and background characteristics, Afghanistan (MICS 2022-23).

Background characteristics	Stunting					Severe Stunting				
	Age (0 to 5 months)	Age (6 to 11 months)	Age (12 to 23 months)	Age (24 to 59 months)	Age (0 to 59 months)	Age (0 to 5 months)	Age (6 to 11 months)	Age (12 to 23 months)	Age (24 to 59 months)	Age (0 to 59 months)
**Gender**										
Female	23.9	21.4	38.3	52.0	43.5	11.7	7.6	16.5	25.7	20.7
Male	28.4	26.8	44.1	52.0	45.5	12.7	11.4	20.6	26.6	22.5
*P*	0.013	<0.001	0.001	0.363	<0.001	0.151	0.001	0.001	0.084	<0.001
**Age in months**										
0 to 5					26.3					12.2
6 to 11					24.2					9.6
12 to 17			36.1		36.1			15.7		15.7
18 to 23			46.8		46.8			21.8		21.8
24 to 36				54.0	54.0				27.8	27.8
37 to 59				50.9	50.9				25.3	25.3
*P*			<0.001	<0.001	<0.001			<0.001	<0.001	<0.001
**Birth order**										
First	31.4	25.4	39.2	50.4	43.7	12.5	9.8	17.3	23.6	19.8
2 to 3	23.5	25.7	38.9	50.4	43.0	12.2	10.6	16.4	25.5	20.9
4 or more	26.2	23.0	43.1	53.3	45.7	12.1	8.9	20.3	27.3	22.6
*P*	0.009	0.398	0.011	0.116	0.037	0.144	0.550	0.066	0.024	0.024
**Preceding birth interval**										
First child	31.8	25.6	39.6	50.3	43.8	13.3	10.0	17.6	23.7	20
<2	25.6	28.4	51.2	58.1	52.2	11.4	9.8	24.4	32.4	27.7
>=2	24.8	22.3	37.5	49.0	41.0	12.1	9.4	16.5	23.3	19.1
*P*	0.002	0.001	<0.001	<0.001	<0.001	0.099	0.823	<0.001	<0.001	<0.001
**Age of mother (In Years)**										
15 to 19	31.9	19.7	48.9	73.8	45.2	10.4	11.6	26.5	41.2	23.1
20 to 29	24.7	25.1	40.0	52.9	44.2	11.5	9.0	17.9	26.8	21.4
30 to 39	28.4	23.3	41.5	50.0	44.2	14.1	8.9	18.3	25.4	21.6
40 to 49	20.9	26.2	43.6	52.3	47.4	11.6	15.0	20.5	24.1	22.2
*P*	0.236	0.551	0.024	<0.001	0.041	0.790	0.036	0.013	<0.001	0.599
**Mother educational attainment**										
Uneducated	26.7	26.2	45.2	54.9	47.5	11.7	11.0	21.8	28.8	24.1
Educated	24.8	18.0	28.7	40.3	33.6	13.9	5.1	8.5	15.4	12.6
*P*	0.005	<0.001	<0.001	<0.001	<0.001	0.221	<0.001	<0.001	<0.001	<0.001
**Marital status**										
Currently Married	26.3	24.2	41.3	52.0	44.5	12.2	9.5	18.6	26.2	21.6
Widowed/Divorced/Separated	18.3	20.4	30.2	50.0	45.5	18.3	20.4	18.6	22.1	21.5
*P*	0.798	0.480	0.730	0.259	0.444	0.100	0.788	0.077	0.049	0.873
**Exposure to mass media**										
No	27.3	26.0	46.1	56.4	48.5	12.1	11.0	21.7	29.8	24.6
Yes	23.8	20.0	30.5	41.2	35.1	12.4	6.1	11.8	17.2	14.5
*P*	<0.001	<0.001	<0.001	<0.001	<0.001	0.237	<0.001	<0.001	<0.001	<0.001
**Place of Residence**										
Urban	31.0	17.1	26.6	38.7	33.3	16.4	5.6	10.4	15.9	13.8
Rural	24.9	26.4	45.8	55.7	47.8	11.0	10.7	21.2	29.1	23.9
*P*	0.007	<0.001	<0.001	<0.001	<0.001	0.203	0.005	<0.001	<0.001	<0.001
**Household wealth index quintile**										
Poorest	26.3	33.4	53.7	62.0	54.5	11.1	16.4	27.4	35.3	29.8
Poor	27.3	28.5	49.2	58.9	51.0	11.5	11.0	21.7	31.9	26
Middle	24.1	20.8	43.9	55.7	46.5	9.7	6.7	21.2	28.5	22.9
Rich	26.4	23.9	33.6	45.0	38.5	14.3	9.3	13.5	19.4	16.6
Richest	27.4	13.1	24.3	33.3	28.8	14.7	3.7	8.3	11.6	10.4
*P*	<0.001	<0.001	<0.001	<0.001	<0.001	0.234	<0.001	<0.001	<0.001	<0.001
**Improved drinking water facility**										
Yes	27.0	22.7	38.5	49.8	42.5	12.3	8.6	16.5	24.3	19.9
No	24.2	29.0	48.6	57.9	50.2	11.8	12.6	24.6	31.1	26.3
*P*	0.005	0.002	<0.001	<0.001	<0.001	0.009	0.011	<0.001	<0.001	<0.001
**Improve toilet facility**										
Yes	25.3	22.2	36.4	47.0	40.2	13.5	8.5	15.1	22.1	18.5
No	27.1	26.0	45.5	56.3	48.3	11.1	10.5	21.8	29.6	24.4
*P*	0.083	<0.001	<0.001	<0.001	<0.001	0.808	0.001	<0.001	<0.001	<0.001
**Open defecation**										
Yes	28.1	33.6	52.3	64.0	55.2	10.5	14.4	28.1	36.5	30.1
No	25.7	21.9	38.4	48.7	41.7	12.7	8.3	16.2	23.4	19.4
*P*	0.013	<0.001	<0.001	<0.001	<0.001	0.296	<0.001	<0.001	<0.001	<0.001
**Region**										
Central	30.3	23.5	36.3	43.7	38.9	15.7	8.3	14.2	18.4	16.3
East	26.7	27.1	43.3	55.6	47.3	11.3	10.0	21.6	29.4	24
North	20.0	22.1	38.4	52.3	43.3	9.2	8.6	16.1	26.1	20.6
South	30.1	29.6	54.3	64.2	55.0	12.4	13.8	28.5	37.9	30.9
West	25.4	21.4	41.4	51.9	44.5	11.4	8.5	19.9	26.8	22.3
*P*	0.045	<0.001	<0.001	<0.001	<0.001	0.245	<0.001	<0.001	<0.001	<0.001
**Ever breastfed**										
Yes	26.3	24.2	41.2			12.2	9.6	18.5		
No	23.7	23.7	40.5			13.4	8.8	20.6		
*P*	0.589	0.986	0.283			0.327	0.440	0.258		
**Early initiation of breastfeeding**										
Yes	28.0	22.0	38.4			14.2	8.0	16.3		
No	24.7	26.3	43.8			10.3	11.0	20.7		
*P*	0.571	<0.001	0.007			0.375	0.001	0.006		
**Exclusively breastfed for the first three days after birth**										
Yes	24.6	24.8	40.4			11.5	10.0	18.2		
No	28.8	23.3	42.5			13.3	8.8	19.2		
*P*	0.529	0.135	0.430			0.582	0.326	0.349		
**Exclusive breastfeeding under six months**										
Yes	27.0					12.1				
No	25.1					12.4				
*P*	0.111					0.822				
**Mixed milk feeding under six months**										
Yes	26.0					12.8				
No	26.3					12.1				
*P*	0.198					0.610				
**Minimum dietary diversity 6–23 months**										
Yes		17.2	32.0				7.3	12.6		
No		24.9	43.2				9.8	19.9		
*P*		0.002	<0.001				0.030	<0.001		
**Minimum meal frequency 6–23 months**										
Yes		20.5	40.8				7.4	17.3		
No		26.0	41.4				10.6	19.4		
*P*		0.001	0.434				<0.001	0.016		
**Minimum acceptable diet 6–23 months**										
Yes		19.5	34.0				11.1	14.0		
No		24.4	41.9				9.5	19.0		
*P*		0.201	0.004				0.009	<0.001		
N	26.3	24.2	41.2	52.0	44.5	12.2	9.6	18.6	26.2	21.6

Furthermore, the southern region has the highest prevalence (55·1%, p < 0·05 with Tukey HSD test, S2 Table). Ghor (67·2%), Urozgan (66·0%), and Zabul (61·8%) provinces show the highest prevalence within subnational areas (S3 Table). Socioeconomic factors also play a role, with children from the lowest wealth quintile having a much higher stunting prevalence (54·5%) than the highest (28·8%). Limited access to improved drinking water (50·2%) and sanitation (48·3%) were associated with increased stunting. Early breastfeeding initiation (within one hour) doesn’t consistently correlate with stunting across age groups. However, dietary diversity in children aged 6-23 months significantly impacts prevalence, with those meeting minimum intake norms having a lower rate (29·2%) compared to those who don’t (36·6%, p < 0·01, Wilcoxon rank test). Similarly, children receiving a minimum adequate diet had lower stunting prevalence (31·1% vs. 35·9%).

Severe stunting affects 21·6% of under-five children, with a higher burden among older children (24-59 months: 26·2%) compared to those under two (14·7%, Table 3). Boys (22·5%) exhibited a slightly higher prevalence than girls (20·7%). Inadequate birth spacing (32·4%) and teenage mothers (41·2%) were significant risk factors for severe stunting. Maternal education also played a role, with children of mothers lacking formal education experiencing a prevalence of 24·1%. Limited maternal media exposure (no weekly exposure: 24·6%) correlated with higher severe stunting rates. Geographically, rural areas (23·9%) had a higher burden than urban areas (13·8%). The southern region displayed the highest prevalence (30·9%) of severe stunting, with Urozgan province exhibiting nearly 50% of severe stunting rates (S3 Table). Notably, there was a three-fold increase in the prevalence of severe stunting among the poorest quintile (29·8%) compared to the wealthiest (10·4%). Lack of access to improved drinking water (26·3%) and sanitation (24·4%) significantly increase the risk. Furthermore, open defecation practices within households were associated with a severe stunting prevalence of 30%.

Logistic regression models were used to estimate stunting risk across correlates for three age groups: 0-5 months, 6-11 months, 12-23 months, 24-59 months, and 0-59 months (Table 4). Females (0-59 months) had an 11% lower risk of stunting compared to males [OR 0·89, 95% CI (0·84; 0·95)]. Compared to infants (0-5 months), children aged two years and above displayed a three-fold increased risk of stunting [OR 3·49, 95% CI (3·02; 4·03). Higher maternal education is associated with better child growth, with children of educated mothers having a 22% lower likelihood of being stunted (OR 0.78, 95% CI: 0.71; 0.86) for stunting, compared to children of uneducated mothers. For the overall 0-59 months group, weekly exposure to mass media (radio, television, internet, or newspaper) reduced stunting risk by 14% [OR 0·86, 95% CI (0·79; 0·94)]. Further disaggregation of the age group reveals that the risk of stunting increases significantly during 18-23 months compared to 12-17 months. The adverse effect of higher birth order on stunting is more prominent in the age group 12-23 months. First born children are found to be at greater risk of stunting compared to children with shorter or adequate birth intervals. Role of maternal education in reducing the risk of stunting is found to be more significant after 12 months of age. Exposure to mass media reduces the risk of stunting during the months of exclusive breastfeeding as well as after two years of age. Household wealth quintile has limited influence on stunting among children below one year of age. There is no significant association of wealth quintile with stunting during the first six months. Open defecation emerges as a significant determinant of stunting for children age 2 years and above.

**Table 4:** Logistic regression odds ratios for association of stunting with domain characteristics by age group categories, Afghanistan (MICS 2022-23).

Independent Variable	0 to 5		6 to 11		12 to 23		24 to 59		0 to 59	
	OR	95% CI	OR	95% CI	OR	95% CI	OR	95% CI	OR	95% CI
**Sex**										
Female vs Male (ref.)	0.8	[0.64,1.01]	0.71**	[0.56,0.89]	0.74***	[0.64,0.86]	0.98	[0.90,1.06]	0.90***	[0.84,0.95]
**Age of child (in months)**										
6-11 vs. 0-5 (ref.)									0.91	[0.77,1.08]
12-17 vs. 0-5 (ref.)									1.63***	[1.39,1.91]
18-23 vs. 0-5 (ref.)									2.77***	[2.35,3.25]
24-36 vs. 0-5 (ref.)									3.49***	[3.02,4.03]
37-59 vs. 0-5 (ref.)									3.06***	[2.67,3.51]
18-23 vs. 12-17 (ref.)					1.74***	[1.50,2.01]				
37-59 vs. 24-36 (ref.)							0.89**	[0.82,0.97]		
**Birth order**										
2 or 3 vs. First Birth (ref.)	2.18	[0.44,10.87]	1.6	[0.40,6.48]	6.32**	[1.91,20.96]	0.77	[0.30,2.02]	1.55	[0.73,3.32]
4 or more vs. First Birth (ref.)	2.39	[0.47,12.29]	1.13	[0.27,4.74]	6.74**	[2.00,22.71]	0.87	[0.33,2.28]	1.67	[0.78,3.59]
**Preceding birth interval**										
Less than 2 vs. First Child (ref.)	0.33	[0.07,1.64]	0.65	[0.16,2.60]	0.23*	[0.07,0.75]	1.61	[0.62,4.19]	0.76	[0.36,1.63]
2 or more vs. First Child (ref.)	0.31	[0.06,1.53]	0.47	[0.12,1.88]	0.13**	[0.04,0.45]	1.2	[0.46,3.13]	0.56	[0.26,1.18]
**Mother Age**										
20-29 vs. 15-19 (ref.)	0.87	[0.57,1.32]	1.73*	[1.07,2.79]	0.82	[0.57,1.18]	0.45***	[0.31,0.67]	0.76**	[0.63,0.93]
30-39 vs. 15-19 (ref.)	1.08	[0.65,1.79]	1.83*	[1.04,3.22]	0.8	[0.53,1.20]	0.36***	[0.24,0.54]	0.67***	[0.54,0.83]
40-49 vs. 15-19 (ref.)	0.72	[0.37,1.38]	2.04*	[1.04,3.99]	0.78	[0.49,1.24]	0.36***	[0.24,0.54]	0.66***	[0.52,0.83]
**Mother Education**										
Educated vs. Uneducated (ref.)	0.95	[0.68,1.32]	0.77	[0.55,1.07]	0.72**	[0.59,0.89]	0.78***	[0.69,0.87]	0.78***	[0.71,0.86]
**Exposure to mass media**										
Yes vs. no (ref.)	0.73*	[0.54,0.98]	1.06	[0.79,1.42]	0.85	[0.71,1.03]	0.86**	[0.77,0.95]	0.86***	[0.79,0.94]
**Place of Residence**										
Rural vs. urban(ref.)	0.69*	[0.49,0.98]	1.03	[0.71,1.50]	1.38*	[1.07,1.77]	1.15*	[1.02,1.31]	1.14*	[1.03,1.27]
**Wealth quintile**										
Poor vs. Poorest (ref.)	1.04	[0.74,1.45]	0.83	[0.60,1.16]	0.84	[0.68,1.03]	0.95	[0.84,1.06]	0.92	[0.84,1.01]
Middle vs. Poorest (ref.)	0.88	[0.61,1.25]	0.55**	[0.38,0.79]	0.70**	[0.56,0.88]	0.9	[0.79,1.01]	0.81***	[0.74,0.90]
Rich vs. Poorest (ref.)	0.9	[0.60,1.37]	0.7	[0.47,1.04]	0.52***	[0.40,0.67]	0.67***	[0.58,0.77]	0.66***	[0.59,0.74]
Richest vs. Poorest (ref.)	0.89	[0.52,1.51]	0.38***	[0.21,0.66]	0.44***	[0.31,0.61]	0.49***	[0.41,0.59]	0.49***	[0.43,0.57]
**Safe drinking water sources**										
Yes vs. no (ref.)	1.05	[0.82,1.34]	0.87	[0.68,1.12]	0.87	[0.74,1.01]	0.94	[0.86,1.02]	0.92*	[0.86,0.99]
**Open defecation**										
Yes vs. no (ref.)	1.09	[0.82,1.44]	1.29	[0.97,1.71]	0.9	[0.75,1.09]	1.17**	[1.06,1.29]	1.11*	[1.02,1.20]
**Zone wise**										
East vs. central (ref.)	0.86	[0.57,1.30]	1.07	[0.73,1.57]	0.97	[0.76,1.25]	1.27***	[1.11,1.45]	1.15*	[1.03,1.28]
North vs. central (ref.)	0.56***	[0.42,0.74]	0.84	[0.62,1.13]	0.83*	[0.69,1.00]	1.21***	[1.10,1.34]	1.04	[0.96,1.13]
South vs. central (ref.)	0.89	[0.64,1.24]	1.14	[0.81,1.59]	1.56***	[1.24,1.96]	1.69***	[1.49,1.91]	1.53***	[1.39,1.69]
West vs. central (ref.)	0.72	[0.47,1.09]	0.78	[0.53,1.14]	0.92	[0.71,1.18]	1.11	[0.97,1.28]	1.02	[0.91,1.14]
**Early initiation of breastfeeding**										
Yes vs. no (ref.)	1.32*	[1.04,1.66]	0.82	[0.65,1.03]	0.92	[0.79,1.07]				
**Exclusively breastfed for the first three days after birth**										
Yes vs. no (ref.)	0.74*	[0.58,0.95]	1.15	[0.91,1.45]	1	[0.85,1.17]				
**Minimum dietary diversity 6–23 months**										
Yes vs. no (ref.)			0.66	[0.42,1.03]	0.78*	[0.63,0.96]				
N	3174		3008		5873		19134		31189	

Children in rural areas are 14% more likely to be stunted than their urban counterparts [OR 1·14, 95% CI (1·05; 1·23)]. The southern region carries the highest stunting burden (OR 1·53, 95% CI 1·39-1·69). Children from the wealthiest quintile are less than half as likely to be stunted as those in the poorest (OR 0·50, 95% CI 0·43-0·58). Access to safe drinking water is significantly associated with reduced risk of stunting among children 0-59 months [OR 0·92, 95% CI (0·86; 0·99)]. Open defecation practices increase stunting risk by 17% in the 24-59 month age group. Region-specific logistic regression also provides similar insights regarding the correlates of stunting (S5 Table).

Logistic regression analysis (S6 Table) identifies factors associated with severe stunting. Under-five girls are 12% less likely to be severely stunted than boys (OR 0·88, 95% CI 0·82-0·95). The risk of severe stunting increases with age and is highest in children aged 37-59 months (OR 2·90, 95% CI 2·40-3·50) and among higher birth orders. Children with teenage mothers are at a higher risk of stunting as compared to all other maternal age groups. Children residing in households that lacked weekly media exposure exhibit an elevated risk of stunting compared to their counterparts in households with such exposure (OR 0.83, 95% CI 0.75-0.93). Conversely, maternal education and higher wealth quintiles are protective factors. Geographically, southern Afghanistan experiences a 78% increase in severe stunting risk compared to the central region (OR 1·78, 95% CI 1·59-1·99).

As a sensitivity check, we conducted an econometric analysis of determinants for stunting by stratifying maternal education and wealth index variables, with results presented in S10-S13 Tables. For children with uneducated mothers, open defecation and short birth intervals were associated with increased risk of stunting among those aged 24-59 months. While children of educated mothers showed reduced stunting risk with exposure to mass media, this effect is not observed for children of uneducated women. Additionally, analysis by wealth quintiles (top 40% vs. bottom 60% households) shows that children from poorer households faced increased stunting risk from open defecation, higher maternal age, and increasing child age. In contrast, exposure to mass media reduces stunting risk among children from wealthier households (top 40% wealth quintile). These findings highlight the varying determinants of stunting across socioeconomic and educational groups.

## Discussion

We analyzed the MICS 2023 survey to examine the levels and patterns of childhood stunting in Afghanistan. Five key findings emerged from the study. First, stunting and severe stunting rates are alarmingly high across Afghanistan, with a disproportionate burden in the southern and eastern regions. Provinces like Ghor, Urozgan, and Zabul show the highest prevalence. Second, maternal education and household economic status are critical determinants of stunting and severe stunting outcomes. Third, stunting prevalence increases with age, peaking in 2-5 year olds. Fourth, higher fertility has an adverse effect and increases the risk of stunting among higher birth order children. Finally, children with limited dietary diversity (consuming four or fewer food groups) are more likely to experience stunting and severe stunting.

The study has certain important limitations. The cross-sectional design prevents establishing causal relationships between factors and stunting. Additionally, the survey data has inherent gaps. For example, missing information on maternal health (height, weight, contraception use) and social factors (gender-based violence) limits our ability to explore their combined impact on child growth. Based on food group intake counts, the dietary diversity indicator reflects the variety of foods consumed but doesn’t capture the quality or quantity of nutrients. Some associations may require further investigation using more robust study designs and specific measures to confirm independent and causal effects. We also recognize that stunting is a multi-factoral problem and limited data on prominent environmental factors including infections are not available to understand the potential role of these important variables. This also restricts the ability of the analysis to draw recommendations on these aspects.

A high prevalence of stunting in the southern region of Afghanistan can be associated with contextual factors, including pre-existing poverty post-conflict international isolation, and economic upheavals [12]. The region is also vulnerable to earthquakes and natural calamities [13]. Studies find significant regional variations in maternal and child health service delivery in Afghanistan. For instance, urban areas (such as Nangarhar, Herat, and Kabul) have higher coverage rates, while remote regions (Northern and Central Highlands) register low maternal and child health services coverage [14]. Access to safe drinking water is another primary concern in Afghanistan. Humanitarian assistance should address the critical social needs of these regions’ healthcare, education, and infrastructure.

Afghanistan’s stunting patterns mirror those in other South Asian nations like Pakistan, Bangladesh, and India. All these countries show a direct association of age with increased stunting prevalence [15]. Similar patterns are noted in Ethiopia and Indonesia [16,17]. Reviews suggest higher stunting rates in boys, although patterns might be mixed or insignificant in some African and South Asian regions [18]. This potential disparity could be linked to differing energy requirements for growth between boys and girls, highlighting the complex interplay between energy expenditure and dietary intake [19]. In the context of Sub-Saharan Africa it is argued that a preferential treatment for females is likely because of a higher value on their role in agriculture. However, clinical studies have often outlined the early biological advantage for females whereas males are found to have higher morbidity and mortality levels. However, in absolute terms the levels of stunting prevalence among both boys and girls are very high and therefore it is important that nutrition policies and programmes should cover all children irrespective of their age and sex.

Furthermore, Afghanistan’s low ranking in the global gender inequality index[20] aligns with our findings. Low female empowerment, reflected in limited literacy and family planning access, significantly impacts childhood stunting. Restricted access to higher education further disadvantages girls and women [21]. Studies across South Asia and Africa consistently show a link between higher maternal education and reduced stunting risk [22]. This link likely arises from several factors. Mothers with more education may have better knowledge and resources to implement optimal childcare practices and dietary choices. It is also worth noting that women with better education may also belong to households with higher economic status. Thus the impact of maternal education becomes even more impactful in the presence of various household endowments. In other words, the role of maternal education cannot be fully captured when discussed in isolation with the economic status of the households. Nevertheless, given the low education levels of Afghan women (15-49 years), immediate interventions should focus on knowledge campaigns promoting exclusive breastfeeding, supplementary feeding [23]. Policies should, however, also increase investments in women’s education to harness the long-term benefits.

Early age marriage and restricted autonomy are all demonstrated to have a negative association with child health and well-being in Afghanistan [24]. Our analysis confirms that maternal factors such as teenage marriage and higher fertility levels can elevate the risk of stunting for children with higher birth order. Studies have noted that teenage pregnancies have a negative impact on birthweight and are a critical determinant of growth failure [25]. Such births are also more likely to be pre-term and can be vulnerable to intrauterine growth restriction. Psychological stress experienced by teenage mothers, consequent upon the challenges of early pregnancy and potential disruptions to educational attainment, may further elevate the risk of stunting in their children [26]. The role of adolescent health and its implications for maternal and child health are now receiving greater attention. Notwithstanding the importance of the first 1000 days of life, the poor status of nutrition among adolescent girls in south Asia has been a concern which causes persistency in the level of child undernutrition. Our findings also reveal that the first born children are often at much higher risk of stunting. The experience of Afghanistan is similar to other countries and contexts where the disadvantage of being first born is noted for various negative health outcomes such as neonatal and infant mortality [27]. Besides, a high rate of marriage among adolescents and early childbearing can further increase the risk of prolonged child undernutrition.

Shorter birth intervals, a risk factor for stunting and underweight in under-fives, are likely linked to Afghanistan’s low contraceptive use (among the lowest in the region) and high total fertility rate (5·2 births per woman) [28,29]. The sociocultural outlook and concerns underlying family planning logistics also restrict the effectiveness of birth spacing promotion in Afghanistan. Consecutive pregnancies and lactation periods can deplete mothers’ nutrition, potentially contributing to Afghanistan’s high maternal morbidity and mortality rates [30,31]. Finally, our study highlights the crucial role of dietary diversity in reducing stunting among children aged 6-23 months. Deficiencies in dietary diversity within this age group, as observed in cross-national studies, elevate the risk of stunting and severe stunting [32]. Promoting complementary feeding practices and dietary variety is essential to reduce stunting and its long-term consequences in Afghanistan. The role of mass media is critical and it shows significant impact particularly during the period of exclusive breastfeeding as well as during the age of 2-5 years. However, the effect is more pronounced among the educated mothers. Also, such campaigns on exclusive breastfeeding can yield rich dividends when coupled with interventions to provide specialized nutritious foods [33]. Moreover, breastfeeding interventions should take into account the local contexts for enhancing their effectiveness. Studies have noted that such interventions are not only sensitive to factors such as maternal age, education and household income but also to status of health facilities and support team delivering the interventions [34].

Stunting outcomes are complex multi-factorial phenomenon and there is only limited evidence on role of various important factors. Open defecation is identified as a key environmental variable and variables related to drinking water facility and toilet facility have odds ratio in the expected direction. Some of the effect of the variable related to toilet facility may be captured by open defecation variable. Improved source of drinking water is available with over three-fourth of the households and thus some of the effects may be associated with water use behavior as well as variation in source for drinking water.

Given the exploratory nature of the study, much of these associations are under-explored largely because of limited data and information on these indicators. Nevertheless, proxy indicators of environmental health such as open defecation levels are found to be significant in our analysis. This further highlights that several factors including environmental factors and preventive mechanisms are necessary to address this multi-factoral problem of stunting. It is important that evidence base is built to understand their influence and accordingly these are treated preventively with hygiene practices, anti-parasite medicines, and vaccines. In addition, greater efforts are warranted to strengthen the community outreach services as the population continues to be vastly uneducated and poor. Despite some relative differentials in the socioeconomic status of the population, all these children continue to reside in conditions that are inimical to fundamental provisions for better child health and nutrition services [35]. Understanding stunting and its barriers beyond the conventional factors is the need of the hour as often action on the conventional variables has showed slow progress. It is important to note that the age-based prevalence of stunting often becomes irreversible and the improvements occurring within five years of age are also limited [36]. This fundamentally requires broad-based changes in the living conditions that includes not only economic factors but also environmental and social factors [37,38]. In particular, there is overwhelming evidence that exclusive breastfeeding for children under six months of age is a high impact practice [39]. Although improvements in water and sanitation infrastructure are resource dependent but it is recommended that nutrition policies and programmes in Afghanistan should place high priority on such behavioral interventions that can have high impact on child nutrition. Also, such programmes should draw upon other potential interventions that have demonstrated considerable impact on child nutrition [40]. These includes interventions such as balanced energy protein supplementation, multiple micronutrient intervention, complementary foods along with complementary feeding education, and small-quantity lipid-based nutrient supplements.

## Conclusion

The alarming prevalence of stunting and severe stunting in Afghanistan necessitates nationwide improvements in nutrition support services. Timely and systematic analysis of levels and determinants based on the latest data can provide much-needed insights for policy action. Our study reveals a high concentration of stunting in Afghanistan’s southern and eastern regions. Notably, stunting outcomes are significantly associated with the child’s demographic, maternal, and household characteristics. Maternal education and household economic status are significant determinants of stunting prevalence. Interventions focusing on maternal health, well-being, and dietary support can substantially improve child nutrition in Afghanistan. However, it is important that the stunting is understood as a multi-dimensional problem and the role of environmental factors that are associated with infections and parasitic diseases are examined for effective preventive measures. Furthermore, improvements in women’s education, adolescent health, community health services for maternal and child health and investments in environmental factors such as safe water and sanitation can sustain these benefits.

## Key Messages

- Childhood stunting is widespread in Afghanistan. The poorest of the poor households should be the priority populations for nutrition support services as well as social and behavioral change interventions for women and infants in nutrition and hygiene focusing on early prevention of stunting.
- Women’s education when endowed with household resources and health information as well as improved access to maternal health care and family planning services are critical factors for protecting children from growth failures.
- Children with poor dietary diversity are at an elevated risk of stunting and severe stunting. Nutrition programmes should focus on reaching out to all children with adequate dietary supplementation and counseling. Environmental factors such as open defecation are also significant and require further focus on entero-pathological infections and treatment.

## Supporting information

S1 Fig Final analytical sample for the study, Afghanistan (MICS 2022-23).
(TIF)

S1 Table Wilcoxon rank test for association of stunting prevalence by age group, Afghanistan (MICS 2022-23).
(XLSX)

S2 Table Tukey HSD test for association of stunting prevalence by age group, Afghanistan (MICS 2022-23).
(XLSX)

S3 Table Prevalence of stunting and severe stunting by Province and age group, Afghanistan (MICS 2022-23).
(XLSX)

S4 Table Regression model selection for stunting based on information criteria, Afghanistan (MICS 2022-23).
(XLSX)

S5 Table Logistic regression odds ratios for association of stunting with domain characteristics by region (0-59 months), Afghanistan (MICS 2022-23).
(XLSX)

S6 Table Logistic regression odds ratios for association of severe stunting with domain characteristics by age group categories, Afghanistan (MICS 2022-23).
(XLSX)

S7 Table Logistic regression odds ratios for association of severe stunting with domain characteristics by region (0-59 months), Afghanistan (MICS 2022-23).
(XLSX)

S8 Table Stepwise Logistic regression odds ratios for association of stunting with domain characteristics by age group, Afghanistan (MICS 2022-23).
(XLSX)

S9 Table Stepwise Logistic regression odds ratios for association of severe stunting with domain characteristics by age group, Afghanistan (MICS 2022-23).
(XLSX)

S10 Table Logistic regression odds ratios for association of stunting with domain characteristics by uneducated women (0-59 months), Afghanistan (MICS 2022-23). (XLSX)

S11 Table Logistic regression odds ratios for association of stunting with domain characteristics by educated women (0-59 months), Afghanistan (MICS 2022-23).
(XLSX)

S12 Table Logistic regression odds ratios for association of stunting with domain characteristics by Bottom 60 of household wealth quintile (0-59 months), Afghanistan (MICS 2022-23).
(XLSX)

S13 Table Logistic regression odds ratios for association of stunting with domain characteristics by Top 40 of household wealth quintile (0-59 months), Afghanistan (MICS 2022-23). (XLSX)
