# Supplementary figures and images for: Levels, severity, and determinants of stunting in children 0–59 months in Afghanistan: Secondary analysis of Multiple Indicator Cluster Survey, 2022-23
Source: PLOS Glob Public Health. 2025 Apr 8;5(4):e0004423. doi: 10.1371/journal.pgph.0004423 (PMC11978115; doi:10.1371/journal.pgph.0004423)

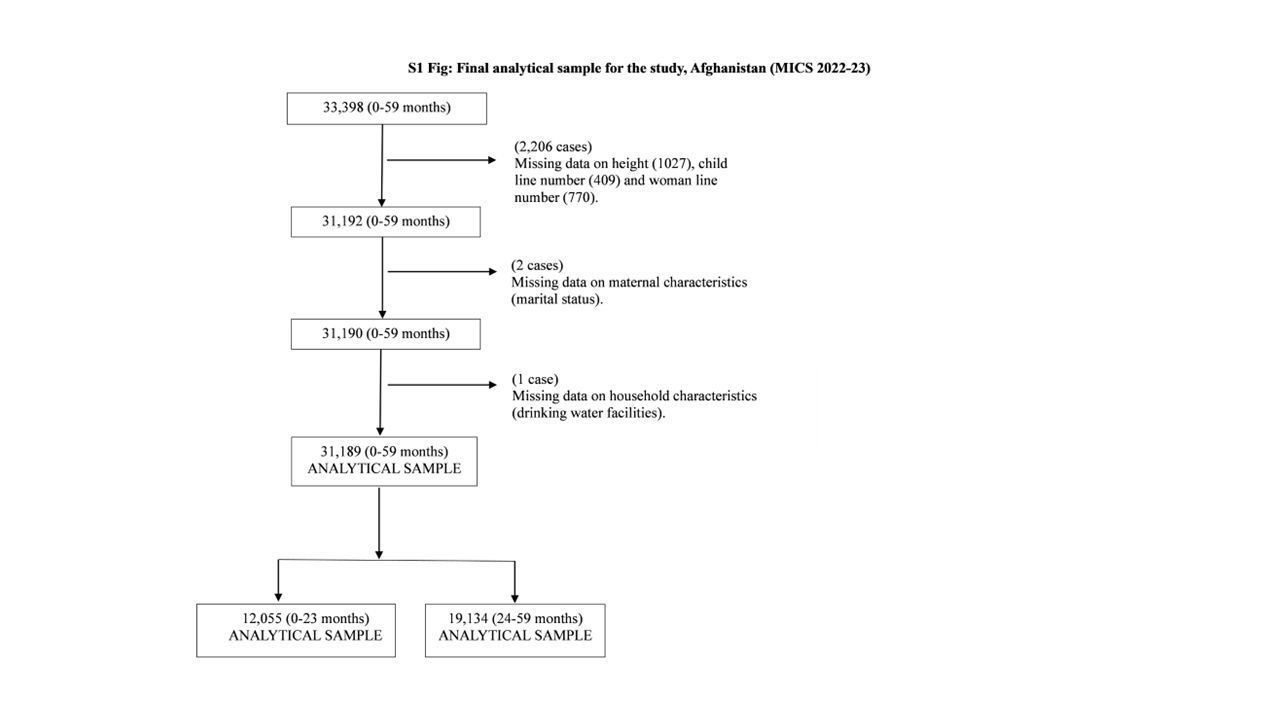

Supplement: S1 Fig — (TIF) [file pgph.0004423.s001.tif]
